# The Physician as a Temperance Reformer*Read before the Parker and Palo Pinto County Medical Societies in joint session, Weatherford, Texas, December 15, 1908.

**Published:** 1909-08

**Authors:** Alexander S. Garrett

**Affiliations:** Springtown, Texas


For Texas Medical Journal.

### The Physician as a Temperance Reformer.*

BY ALEXANDER S. GARRETT, M. D., SPRINGTOWN, TEXAS.

*Mr. Chairman and Gentlemen:*

The only apology I offer for attempting to read a paper on the above subject before an intelligent body of physicians is my deficiency in scientific attainment and limited experience in treating diseases that were caused by drinking alcoholic drinks. I trust, however, that realizing the magnitude and importance of the subject you will overlook any unavoidable deficiencies on my part.

If I believed that the first and only duty of the physician is the treating of the sick and rendering medical and surgical assistance to the injured, no time or thought would be given to a subject of this kind. But, gentlemen, is not the medical profession stirred today from center to circumference as never before with the all-important, and, I believe, Divine idea, of preventive medicine; the teaching the world how to keep well and prevent people from getting sick? This being true, preventive medicine being the highest sphere of the physician's work, and alcohol being so intimately connected and interwoven with so many diseases, is one reason why I have chosen this subject. But because alcohol produces disease is not the only reason why doctors should be temperance reformers. Moral reasons, the demand for good citizenship, peace, love, happiness and prosperity also appeal to the conscientious doctor and ask him to join in the lead for temperance reformation.

Doctors are teachers of the people in all things pertaining to their physical health. The minister sees the terrible consequences resulting to the moral nature of man from indulgence in alcohol and cries aloud against the saloon and drunkenness. The physician sees not only the moral nature destroyed, but he sees the physical manhood and womanhood wrecked and ruined from the poisonous effects of alcohol, and weakness and diseases of numerous kinds visited on the helpless child on account of the sins and mistakes of the parents before the child was born.

---

*Read before the Parker and Palo Pinto County Medical Societies in joint session, Weatherford, Texas, December 15, 1908.

Dr. Benjamin Ward Richardson, of London, England, in his work on "Diseases of Modern Life," writing of alcohol, says:

"To have to speak of diseases originating from the use of a fluid which, next to water, forms a part of the daily beverage of immense populations of civilized peoples, seems a satire on civilization. It is, nevertheless, the duty of every physician to speak plainly on this subject, because it his painful task, day by day, to treat the most terrible and fatal diseases, for the origin of which he can assign no other cause than the use of alcohol."

Fellow physicians, as we help spread the glorious news to the people at large as it comes from our greatest and most scientific men in the medical profession, that consumption or tuberculosis is a contagious and preventable disease, let us at the same time teach them that alcoholism leads to the development of a very severe and fatal form of consumption, and that alcohol is not a preventive of consumption nor a remedy for it, neither is alcohol in any form a preventive of *any* disease and only a remedy for a few symptoms or conditions that may arise in some cases of actual or real sickness.

At a recent meeting of the American Society for the Study of Inebrity and Alcoholism in Chicago, Dr. D. H. Brewer, in a paper on the "Prevalence of Alcohol as an Active and Predisposing Cause of Brain and Nervous Diseases," asserted "that all the most advanced studies in the etiology of neurotic diseases made alcohol more and more prominent, and, next to syphilis, may be considered the greatest active cause of disease in modern civilization."

The Connecticut Hospital for the Insane reports that during the last two years nearly one-third of all cases admitted gave alcohol as the etiological factor. So states the summer number of the *Journal of Inebriety,* edited by Dr. T. D. Crothers, Hartford, Conn.

A short time ago I wrote Dr. B. M. Worsham, late Superintendent of the Insane Asylum at Austin, for a statement of the per cent of cases received into the Austin Asylum in which the cause was due either directly or indirectly to alcohol. He replied by stating that no record of the causes of insanity had been kept, but that he supposed 18 or 20 per cent was due to alcohol. This was a low estimate, according to many other reports I have seen given by hospital or asylum men. In 577 epileptics received at the Craig Colony Hospital, at Sonyea, New York, last year, 259 showed a history of inebriety and alcoholism in the parents. (*The Journal of Inebriety,* Summer Number.)

Dr. Podstadt, Superintendent of the Southern Illinois Insane

Asylum at Elgin, Illinois, says: "There are tbout 8000 epileptics in the State, more than half of whom are children. Of these nearly two thousand could, if they knew enough, just point their fingers at their father or mother or both and say: 'You are responsible for my misery. You through the alcohol which made you its slave.'"

That which is true in New York and Illinois is true of Texas or any other country where alcoholic drinks are used in reference to the cause of epilepsy.

A few weeks ago I write to the Superintendent of the State Epileptic Colony at Abilene inquiring of him the per cent of cases admitted in which the cause was known to be directly or indirectly alcoholic.

He replied by stating that "We have no patients in the Colony whose affliction can be traced *directly* to the abuse of alcohol by the patients themselves; that the history of the patients is hard to get at accurately; still, there is no doubt that many of the defective class of the population of the State have had ancestors addicted to alcohol. Heredity plays a more important part in the production of epilepsy than almost any other cause."

I think we can safely say that it is a well established fact that a large per cent of all cases of insanity and epilepsy are of alcoholic origin.

The undisputed fact that alcohol causes consumption, insanity and epilepsy is sufficient to make every thinking physician a strong advocate of temperance reform. But, gentlemen, you all know that this is not the half.

Gentlemen, you all know that hundreds and thousands are dying every year in our country from the poisonous effects of alcohol who never saw an insane asylum or an epileptic colony.

How about some of the lawyers, and the doctors, and the business men whom we have all known who have gone to their graves prematurely from the effects of alcohol when they should have been living lives of usefulness to their families and to their country?

We can not afford to keep silent on this important subject. We can not afford to leave this great work of reformation entirely in the hands of the ministry. It is too great and too important for us as physicians to keep our tongues and our pens silent in regard to it.

There are scarcely any of the delicate tissues of the human body that escape the hardening, degenerative, and destructive effects of alcohol when taken into the system.

Prof. W. S. Hall, of Chicago, in a paper on "Recent Researches in the Physiology of Alcohol on the Cells and Tissues," concludes "that laboratory research work from every point of view confirmed the statement that alcohol was a paralyzant and its action on the cells and tissues was corroding and destructive, both chemically and physiologically."

Dr. T. D. Crothers, Superintendent Walnut Lodge Hospital, Hartford, Connecticut, and editor of *The Journal of Inebriety,* in in a paper on "Autointoxication From Beer and Spirits," says: "The newer knowledge concerning the action of alcohol shows that instead of aiding digestion it impairs it, and that it may be the cause of serious autointoxication."

After discussing the subject at some length and giving numbers of illustrative cases, he concludes that "Alcohol in any form taken into the body as a beverage, is not only a poison, but produces other poisons, and associated with other substances it may develop toxins. Alcohol is also an anesthetic and not a tonic or so-called stimulant. It increases the waste products of the body and diminishes the power of elimination. It also destroys the phagocytes of the blood and thus removes and lessens the protective power of the blood cells."

Many of you gentlemen can call to mind no doubt cases of diseased stomachs, livers, kidneys, hearts, blood vessels and nervous systems that you have been required to treat with little satisfaction to yourselves and still less to the patient because alcohol had done its work too surely and securely to be overcome.

Dr. G. Frank Lydston, of Chicago, says that "moral persuasion will not restore a gin liver or kidneys to a normal condition." He might have said with equal propriety that medicine will not restore the damage either.

In the same paper in which he discusses "Inebriety and Its Relation to Crime" he says that "fully 70 per cent of all crimes of impulse, brutality and emotion are committed by persons whose brains are alcoholized."

He says that "the rise and fall of crime in Chicago has been found to correspond with the privileges accorded the all-night saloon."

The sad fact stares us in the face that recent observations and studies in alcohol and its effects on the human system teach us that the moderate drinker is in greater danger than the one who takes an occasional spree and then quits. The moderate drinker is playing with a fire that will certainly sooner or later consume him and he knows it not.

Dr. Lewis D. Mason, Brooklyn, N. Y., Vice-President of the American Society for the Study of Inebriety and Drug Neurosis, says: "It may be regarded as a scientific axiom and a medical fact that under no conditions, under no limitations, is the habitual or moderate use of alcohol safe, and any person or physician who so recommends the use of alcohol habitually as a beverage or for medical purposes, has advised the first step toward physical, mental and moral degeneracy."

Under the head of hereditary influence he says: "In the study of 600 cases that came under my supervision, in which I made a study of the family history, none escaped the record of antecedent degeneracy, from various forms of narcomania, nervous diseases, consumption, and other conditions of alcoholic degeneration, evidence enough to demonstrate the relative sequence between drinking parents and a drunken posterity."

On the same line of thought Dr. Benjamin Ward Richardson, London, from whom I first quoted, writing of the vices that are inherited from parents, says: "But not one of the transmitted wrongs, physical or mental, is more certainly passed on to those yet unborn than the wrongs which are inflicted by alcohol."

If people were to quit drinking whisky, beer, brandy, the pure alcohol itself or any other stuff that contains it, now, it would take several generations to overcome the bad effects that have already been done to the human race.

Probably the worst effects of alcohol is produced on women. It is bad enough for men to drink, but when women in high society as well as the unfortunate strumpet that walks the street at night, drink, it is simply awful to think about. There must be a check or else our nation will go down as did the inhabitants of the rich valley of Samaria before the conquering tread of Assyrian host.

Does whisky and beer drinking produce disease? Do physicans want to advocate the saloon and whisky drinking from a mercenary standpoint? Of course they do not. No physician who is worthy the name could offer to do that. Our profession is too high, too noble, too great. Our highest duty is to teach mankind and help relieve the world from disease and the cause of disease.

If I would not offend the kind-heartedness and good intentions of my fellow physicians who arranged to have a "smoker" in connection with our program tonight, I would say that doctors ought also, to take the lead and stand erect as men, facing the public by example as well as by precept in the great reformation now going on to free the world from the tobacco curse as well as from the alcohol curse.

Fellow physicians, I might weary you if I continued further on this subject. It is so great, so important and demands so much of our attention, our sympathy, our help and our very best labors and thought that I know not where to stop. When we get hold of the truth let us pass it on and help our fellowman.

Tell it on the hills and mountains, tell in the plains and valleys, tell it in the timbers and tell it on the prairies, tell it in the towns and tell it in the cities, tell it in the homes and tell it everywhere, tell it with your tongue and tell it with your pen, that alcohol is a poison, a curse and a destroyer of human happiness and human life.

"Softly breaks the morning light
O'er the peaceful slumbering earth,
Banishing the gloom of night,
Waking all things into mirth.

Rosy beams illume the hills,
Then descending, valleys glow;
Now no cloud of darkness fills
Any spot of earth below.

Thus the truth in silent pow'r
Dawns upon the human brain,
Touching first the heights that tower,
Then, expanding, floods the plain."

---

When gas comes from an abscess which has been opened in some part of the abdomen, it must not be hastily assumed that the bowel is involved, as many of the abdominal suppurations are associated with gas-forming bacteria. This is notably the case with subphrenic abscesses.—*American Journal of Surgery.*

